# Methylation Microarray Studies Highlight *PDGFA* Expression as a Factor in Biliary Atresia

**DOI:** 10.1371/journal.pone.0151521

**Published:** 2016-03-24

**Authors:** Zenobia C. Cofer, Shuang Cui, Steven F. EauClaire, Cecilia Kim, John W. Tobias, Hakon Hakonarson, Kathleen M. Loomes, Randolph P. Matthews

**Affiliations:** 1 Division of Gastroenterology, Hepatology, and Nutrition, The Children’s Hospital of Philadelphia Research Institute, Philadelphia, Pennsylvania, United States of America; 2 Center for Applied Genomics, The Children’s Hospital of Philadelphia, Philadelphia, Pennsylvania, United States of America; 3 Department of Pediatrics, Perelman School of Medicine, University of Pennsylvania, Philadelphia, Pennsylvania, United States of America; 4 Penn Center for Biomedical Informatics, University of Pennsylvania, Philadelphia, Pennsylvania, United States of America; University of Medicine, Greifswald, Germany, GERMANY

## Abstract

Biliary atresia (BA) is a progressive fibro-inflammatory disorder that is the leading indication for liver transplantation in children. Although there is evidence implicating genetic, infectious, environmental, and inflammatory causes, the etiology of BA remains unknown. We have recently reported that cholangiocytes from BA patients showed decreased DNA methylation relative to disease- and non-disease controls, supporting a potential role for DNA hypomethylation in BA etiopathogenesis. In the current study, we examined the methylation status of specific genes in human BA livers using methylation microarray technology. We found global DNA hypomethylation in BA samples as compared to disease- and non-disease controls at specific genetic loci. Hedgehog pathway members, *SHH* and *GLI2*, known to be upregulated in BA, were both hypomethylated, validating this approach as an investigative tool. Another region near the *PDGFA* locus was the most significantly hypomethylated in BA, suggesting potential aberrant expression. Validation assays confirmed increased transcriptional and protein expression of PDGFA in BA livers. We also show that PDGF-A protein is specifically localized to cholangiocytes in human liver samples. Injection of PDGF-AA protein dimer into zebrafish larvae caused biliary developmental and functional defects. In addition, activation of the Hedgehog pathway caused increased expression of PDGF-A in zebrafish larvae, providing a previously unrecognized link between PDGF and the Hedgehog pathway. Our findings implicate DNA hypomethylation as a specific factor in mediating overexpression of genes associated with BA and identify PDGF as a new candidate in BA pathogenesis.

## Introduction

Biliary atresia (BA) is a progressive fibro-inflammatory disorder that is the leading indication for liver transplantation in children. Although there is evidence implicating genetic [1], infectious [2], environmental [3], and inflammatory [4] causes, the etiology of BA remains unknown. Several groups have examined gene expression changes in livers from BA patients [5, 6], noting an increase in imprinted genes [6], supporting a possible role for altered DNA methylation in patients with BA. Importantly, changes in DNA methylation can be elicited by several agents, including viruses [7, 8] and environmental factors [9], suggesting that altered DNA methylation may be an important pathogenic feature in BA, regardless of the proximal cause.

In general, large-scale studies of methylation have focused on cancer and environmental exposures. However, there is increasing evidence for the importance of epigenetic influences on a variety of disorders. Recent studies have examined differential methylation in obese adolescents [10] and in patients with ulcerative colitis [11], demonstrating that there are methylation changes in specific genes in a variety of diseases. While these changes may not reflect the complete pathogenic picture, it seems likely that differential gene methylation does play a role in disease pathogenesis for many conditions.

Zebrafish have emerged as a powerful tool for liver disease modeling [12, 13], and we have reported several zebrafish models with biliary defects. Some of these models share features of BA, such as intra- and extrahepatic biliary defects and activation of pathways known to be important in BA such as Hedgehog (HH) and interferon-gamma (IFNγ). Recently we reported that inhibition of DNA methylation in zebrafish led to increased expression of *ifng* genes in zebrafish hepatocytes [14] and that forced expression of IFNγ can directly lead to biliary defects [15]. Moreover, we noted that cholangiocytes from BA patients showed decreased DNA methylation relative to disease- and non-disease controls, supporting a potential role for DNA hypomethylation in the etiopathogenesis of BA. Others described DNA hypomethylation in lymphocytes of patients with BA, and an increase in expression of IFNγ genes [16]. These studies support DNA methylation as a potential contributor to BA pathogenesis.

In this report, we provide further evidence supporting global DNA hypomethylation in BA patients. Using a methylation microarray study of livers from patients with BA, we examined the methylation status of genes previously identified as upregulated in BA, previously reported as being important in BA pathogenesis, and also identified specific genes previously unknown to be important in BA. We show *PDGF* to be hypomethylated in BA compared to non-disease controls. We also demonstrate that injection of PDGF-AA peptide into developing zebrafish larvae leads to biliary defects and that liver samples from BA patients have increased PDGF-AA protein expression. Additionally, overexpression of HH signaling in fish larvae caused increased expression of PDGF-AA, indicating that PDGF-AA may function as a downstream target in the liver. Our studies underscore the potential importance of DNA hypomethylation and HH signaling in the pathogenesis of BA.

## Materials and Methods

The work in this manuscript involving human participants was approved by the Institutional Review Board at The Children's Hospital of Philadelphia. Prior to collection of liver tissue, written consent was obtained from the parent or guardian of each subject, in accordance with the IRB-approved protocol. All animal work in this manuscript was approved by the Institutional Animal Care and Use Committee at The Children's Hospital of Philadelphia (IAC 14–000850).

### Patient livers and DNA extraction

Liver samples used in this study were taken from liver explants obtained at the time of liver transplantation. Each subject had previously undergone a Kasai hepatoportoenterostomy. Explanted tissue was immediately frozen in liquid nitrogen, and stored in de-identified containers, in accordance with a protocol approved by the CHOP institutional review board. Prior to collection of liver tissue, written consent was obtained from the parent or guardian of each subject, in accordance with the IRB-approved protocol. Liver tissue was homogenized and DNA was extracted using DNeasy (Qiagen). In preparation for the methylation microarray studies, DNA was treated with sodium bisulfite using EpiTect (Qiagen) as per the manufacturer’s protocol. Age, sex, and diagnosis of patients used in the methylation microarray and subsequent studies are listed in S1A and S1B Table.

### Methylation microarray (Illumina Infinium^™^ assay)

At the Center for Applied Genomics at CHOP, we performed the same methods for the HumanMethylation 450 Beadchip as we have for the SNP genotyping beadchips using Illumina Infinium^™^ II BeadChip technology [17, 18] (Illumina, San Diego). We used 500 ng of bisulfite-converted DNA to genotype each sample, according to the manufacturer’s guidelines. On day one, the converted DNA was amplified 1000-1500-fold. Day two, amplified DNA was fragmented ~300–600bp, then precipitated and re-suspended followed by hybridization on to a BeadChip. Single base extension utilizes a single probe sequence ~50bp long designed to hybridize immediately adjacent to the SNP query site. Following targeted hybridization to the bead array, the arrayed SNP locus-specific primers (attached to beads) were extended with a single hapten-labeled dideoxynucleotide in the SBE reaction. The haptens were subsequently detected by a multi-layer immunohistochemical sandwich assay, as described [17, 18]. The Illumina iScan scanned each BeadChip at two wavelengths and created an image file. As BeadChip images were collected, intensity values were determined for all instances of each bead type, and data files were created that summarized intensity values for each bead type. These files consisted of intensity data that was loaded directly into Illumina’s genotype analysis software, GenomeStudio. A bead pool manifest created from the LIMS database containing all the BeadChip data was loaded into GenomeStudio along with the intensity data for the samples. BeadStudio used a normalization algorithm to minimize BeadChip to BeadChip variability. Once the normalization was complete, the clustering algorithm was run to evaluate cluster positions for each locus and assign a beta value (β) for each CpG locus.

For the conglomerate analysis, we took the ratio of beta values for each probe of averaged BA samples over the combined control samples (DC and NDC), and log2 transformed the ratio. This list of all ratios was filtered to include only those probes with an uncorrected p<0.05 by ANOVA comparing the same groups. This list was further filtered to include only those probes that had a log2 (BA/C) of >0.263 or <-0.263, corresponding to a fold change of ~1.2x.

### Quantitative PCR

For gene expression studies, RNA was isolated from explanted livers obtained in the same manner as described above, in accordance with an active IRB protocol. The RNA was isolated using RNeasy (Qiagen) and cDNA was synthesized in accordance with standard procedures. Primers for *PDGFA*, *ARHGEF10*, *ZEB2*, and *ADAP1* are listed in S2 Table, as are primers for *HPRT*, which was used as a normalization control. Quantitative PCR was performed as previously described [1], in technical quadruplicate, with representative samples from non-disease control (NDC), disease control (DC), and BA. Results shown are representative experiments from a total of at least three trials per primer set.

### Immunostaining and western blots

Sample slides were obtained from biopsy specimens from CHOP under an active IRB protocol. Samples included biopsy specimens from NDC, DC, and BA patients, not overlapping with patients used for the molecular studies. Slides were prepared as per standard procedures and stained with anti-PDGF primary antibody (Abcam, ab38562, 1:250) and appropriate secondary antibody.

For western blotting of zebrafish larval samples for PDGF-A: Protein was extracted from thirty pooled 5 days post fertilization (5 dpf) larvae and homogenized in a 10% sodium dodecyl sulfate denaturing medium following standard protocols. Blots were exposed to primary antibodies against PDGF-A (Abcam, ab38562) at a titration of 1:250 or Tubulin (Cell Signaling, 2125) at a titration of 1:1000. Band visualization was conducted using appropriate HRP-conjugated secondary antibodies and standard ECL protocols.

### Zebrafish studies and protein injection

Wild-type Tupfel long fin (TLF) strain zebrafish were raised in accordance with standard practice under a protocol approved by the Institutional Animal Care and Use Committee at The Children's Hospital of Philadelphia. PDGF-AA, PDGF-AB, and PDGF-BB peptide (ProSpec Bio) were injected into the yolk of 2 dpf larvae at a concentration of 25 ug/mL, similar to our previous studies [1]. For HH pathway overexpression studies, larvae were incubated in E3 containing 30μM purmorphamine (Sigma, SML0868) from 2 dpf to 5dpf. At 5dpf, PED-6 was added to E3 for gallbladder uptake assays. Larvae were then sacrificed and fixed, similar to previous studies [19]. Immunostaining using cytokeratin antibody Ks18.04 was performed as in previous studies [20, 21], and the livers were analyzed using confocal microscopy. PED-6 gallbladder uptake was quantified using Chi-square analysis, and duct attributes were quantified as per previous studies and compared using t-test.

## Results

### Methylation microarray analysis of BA patients underscores importance of Hedgehog signaling in BA

We have previously shown that cholangiocytes from patients with BA have decreased DNA methylation [14], and others have shown that there is decreased DNA methylation in lymphocytes from patients with BA [16, 22]. To determine whether DNA methylation of specific genes is decreased in BA livers, we examined global liver DNA methylation from samples taken at transplant. We hypothesized that analysis of methylation changes in the whole liver may uncover hypomethylated genes in BA patients, as we could screen for methylation differences between BA and control patients and perform subsequent studies in earlier specimens to identify cell specificity.

**Fig 1:**
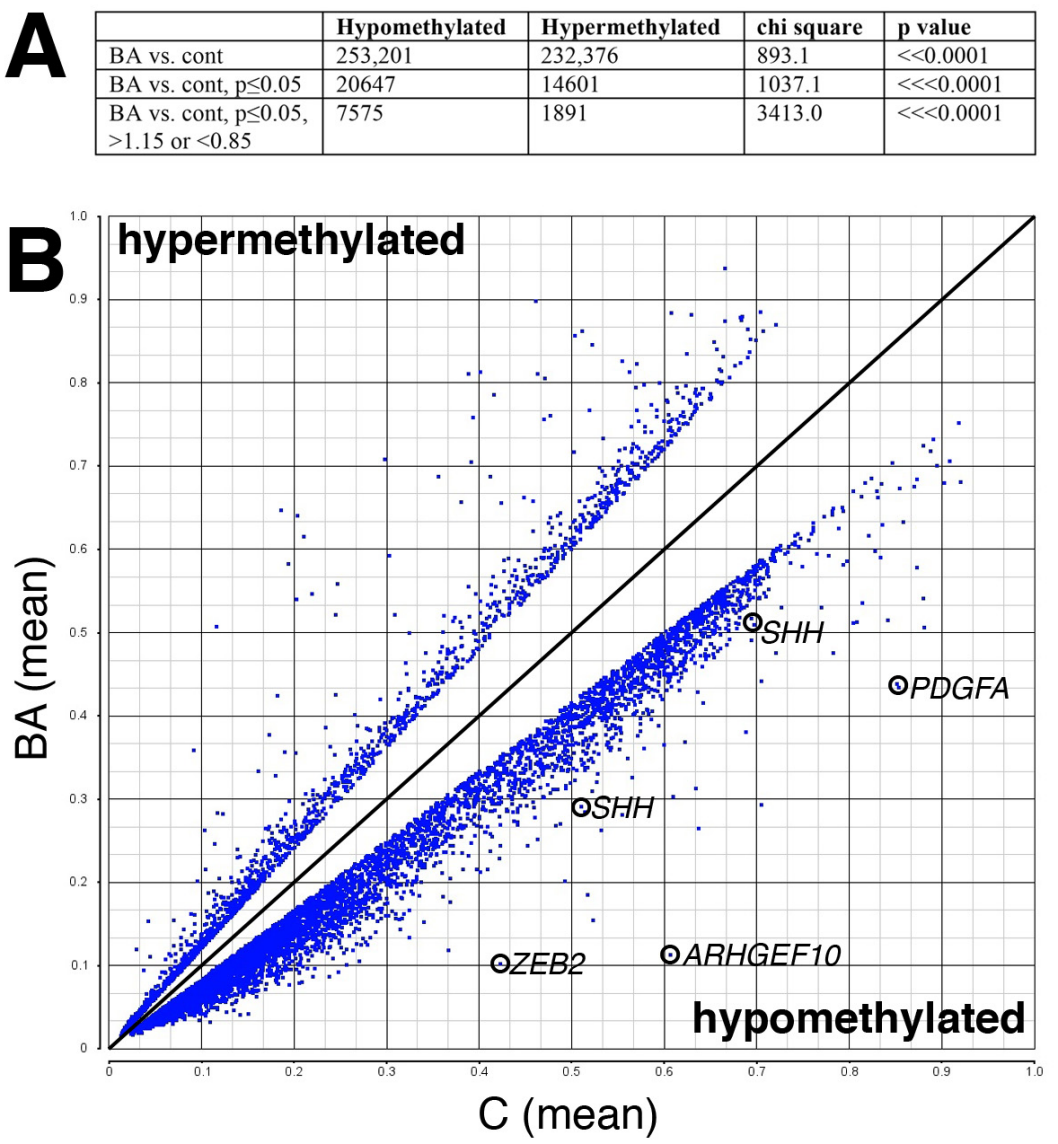
DNA hypomethylation in BA samples. (A) Summary of the data, showing the difference in methylation for all points, for those with statistical significance. The Chi-square value for each comparison and the corresponding p-value, although all p-values were considerably less than 0.0001. (B) Shown is a scatter plot where probe sets are plotted for the relative amount of methylation in control (C, x-axis) and BA (y-axis). The diagonal line represents equal methylation, so points below are relatively hypomethylated in BA, while those above the line are hypermethylated. Only those points with greater than 15% difference and a statistically significant (p<0.05) difference are shown.

We performed methylation microarray analysis on DNA samples from 3 non-disease control (NDC), 4 disease control (DC), and 6 BA liver specimens (S1A Table). Analysis of differential methylation at the 485,585 distinct sites using the Illumina HumanMethylation 450 BeadChip demonstrated significantly more sites with decreased methylation in the BA samples (Fig 1a). This supports our previous studies showing decreased DNA methylation in BA livers, and suggests that specific genes may be hypomethylated in BA.

To identify specific hypomethylated genes in the methylation microarray, we adopted two approaches. First, we perused the list of probesets for genes known to be upregulated in BA patients, based on previous studies. Then, we undertook an unbiased approach and selected probesets with significant differences in methylation from the list of all probes (Table 1). Corrected and uncorrected signal values for all probesets are listed at GEO repository (GSE69948).

**Table 1:** Most significantly hypomethylated targets identified in the microarray, with most closely associated gene(s).

Target	Gene	NDC meth	DC meth	BA meth
cg15567368	*PDGFA*	0.89±0.01	0.83±0.14	0.43±0.27***
cg16748433	*ARHGEF10*	0.55±0.37	0.65±0.36	0.11±0.01***
cg26995506	*ZEB2*	0.47±0.36	0.38±0.24	0.10±0.02**
cg19665696	*ADAP1*	0.53±0.24	0.51±0.22	0.18±0.05***
cg15028160	*PPFIA3*	0.41±0.03	0.34±0.17	0.12±0.02***
cg24131442	*ZC3H12D*	0.57±0.17	0.54±0.14	0.28±0.18**
cg26813483	*C13orf16*	0.58±0.43	0.69±0.18	0.31±0.24*
cg15897435	*MRPL1*	0.59±0.39	0.67±0.13	0.31±0.21*
cg17491987	*TACC1*	0.44±0.10	0.31±0.14	0.18±0.04**
cg01889574	*CD82*	0.51±0.19	0.46±0.15	0.27±0.08**
cg24117274	*RAP1GAP*	0.45±0.08	0.37±0.12	0.22±0.04***
cg24127639	*MYEOV*	0.50±0.17	0.48±0.17	0.27±0.09**
cg04741284	*PMEPA1*	0.46±0.14	0.40±0.17	0.23±0.03**
cg03265486	*IGSF9B*	0.31±0.15	0.23±0.09	0.12±0.02**
cg14928057	*INCA1*	0.40±0.12	0.33±0.12	0.21±0.04**
cg13092766	*BCL2L11*	0.32±0.16	0.31±0.13	0.14±0.02**
cg19045970	*HLA-A*	0.33±0.10	0.31±0.12	0.15±0.05***
cg18197146	*FRS3;PRICKLE4*	0.28±0.12	0.23±0.12	0.12±0.02**
cg08818195	*FBRSL1*	0.30±0.06	0.28±0.13	0.14±0.02***
cg25508118	*STEAP3*	0.25±0.13	0.27±0.13	0.10±0.01**

***p≤0.005,

**p≤0.03,

*p≤0.05, relative to total control.

All targets were also significantly different from DC alone.

**Fig 2:**
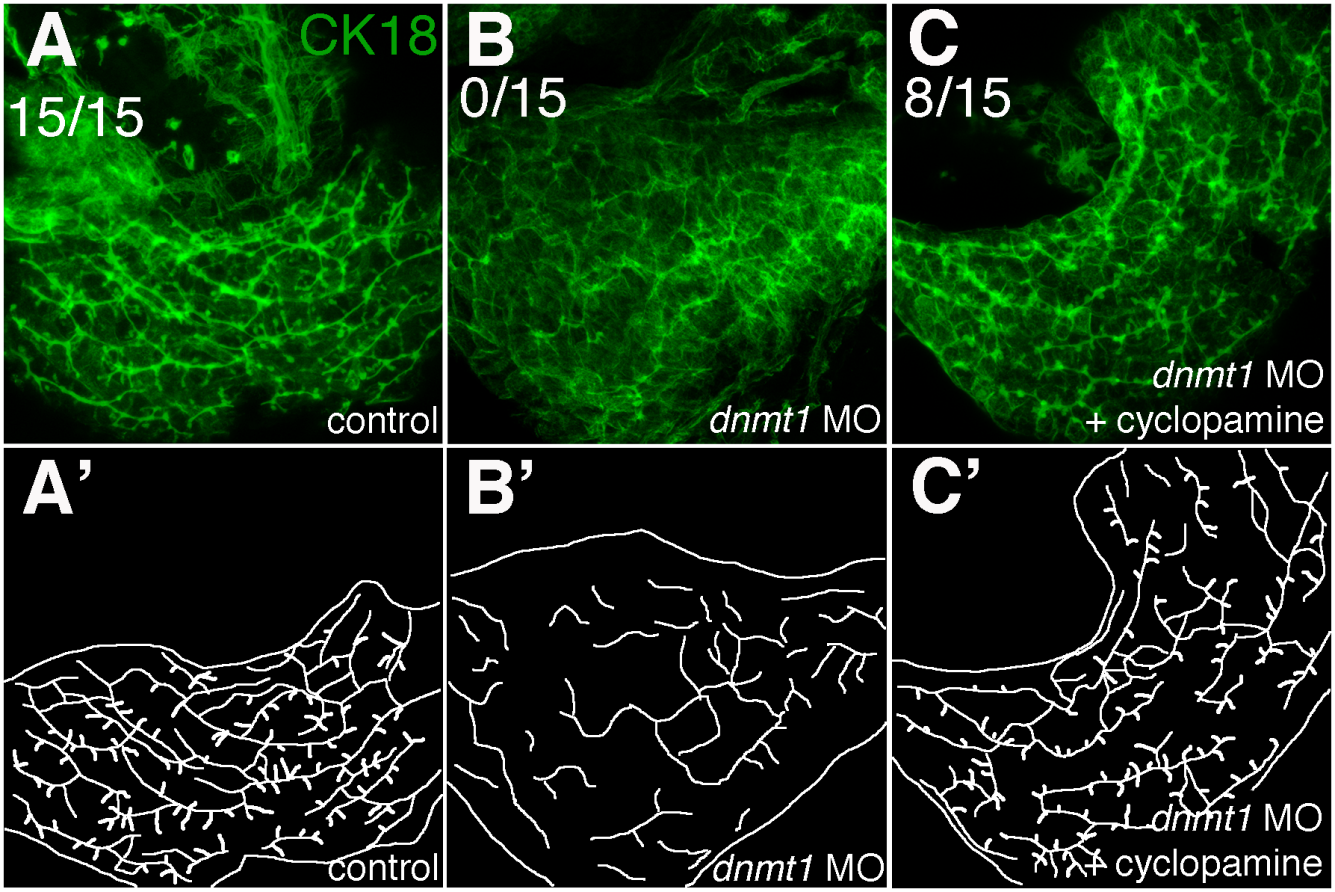
Inhibition of HH signaling rescues liver morphology in DNA hypomethylated larvae. Cytokeratin-18 (green) labels biliary ducts. DNA *methyltransferase* (*dnmt1)* morphants show complete abrogation of biliary structure (B). Incubation of larvae in cyclopamine protects against liver degeneration (C). Numbers represent total livers with normal structure. A’-C’ diagram ductular structure of panels A-C, respectively.

Others have documented that Hedgehog signaling is upregulated in BA, and we have shown that inhibition of HH signaling rescues biliary defects in zebrafish models of cholestatic disease [1, 23]. Thus, we looked at Hedgehog genes in the methylation microarray (Table 2). Interestingly, there were several probe sets near the *SHH* locus that were hypomethylated in BA (Fig 1 and Table 2). The HH effector, *GLI2*, also showed some level of decreased methylation, but other Hedgehog pathway genes did not show significant methylation differences between patient groups (Table 2). We have previously shown that oligonucleotide morpholino-mediated knockdown of DNA methyltransferase-1 (*dnmt1)* causes severe biliary defects in zebrafish larvae ([14] and Fig 2). Consistent with DNA methylation affecting HH signaling and mediating biliary defects, inhibition of HH signaling rescued the biliary phenotype caused by knockdown of *dnmt1* (Fig 2). This supports a model in which inhibition of DNA methylation leads to overactivation of HH that then results in biliary defects, and differential methylation of HH pathway genes in our BA methylation microarray is consistent with this mechanism playing a role in BA.

**Table 2:** Methylation of Hedgehog pathway genes.

Gene	HypoMe in MeMA?	NDC meth	DC meth	BA meth	p value BA vs cont	probe
DHH	N					
IHH	N					
SHH	Y	0.71±0.10	0.72±0.13	0.57±0.09	0.02	cg02900995
		0.68±0.08	0.68±0.13	0.53±0.08	0.02	cg06238944
		0.49±0.05	0.50±0.09	0.36±0.06	0.002	cg09079818
		0.48±0.09	0.42±0.16	0.30±0.06	0.02	cg20381798
		0.17±0.03	0.19±0.05	0.11±0.02	0.002	cg02590556
GLI1	N					
GLI2	Y	0.79±0.06	0.76±0.07	0.68±0.06	0.03	cg12080831
GLI3	N					
GLI4	N					
PTCH1	N					
PTCH2	N					
SMO	N					

SHH (Sonic Hedgehog) demonstrates hypomethylation in BA, and of note there are several probes showing decreased methylation in BA for this gene. GLI2 also shows some hypomethylation, but no other Hedgehog genes appear to be hypomethylated in BA.

### Hypomethylation of genes upregulated in BA

Several expression microarray studies have looked for genes important in BA pathogenesis. These include studies performed on livers from BA patient livers, on mice injected with RRV, and on *in vitro* models of biliary development [5, 6, 24, 25]. We examined our microarray data for methylation differences in genes identified as upregulated in these studies. Several upregulated genes were associated with decreased methylation in BA, with some genes having higher magnitude changes in methylation, or decreased methylation in multiple neighboring probesets (S3 Table). Notably, probesets near the *KRT7*, *SMAD3*, and *LFNG* loci demonstrated varying degrees of hypomethylation. *KRT7* encodes for cytokeratin-7, a marker of newly formed bile ducts [26],while *LFNG* expression modulates Notch signaling and SMAD3 is a known transcription factor that regulates TGFβ signaling, which is an important pathway in BA [27].

Several groups have used the RRV mouse model to uncover genes potentially important in BA. These studies have yielded several families of inflammatory genes, including IFNγ [28], IL-12 [29], IL-8 [30], and complement family members [31]. IFNγ pathway activity is important in BA, with studies showing hypomethylation of IFNγ family genes in lymphocytes from BA patients [16, 22]. Thus, we examined methylation of inflammatory genes in our microarray. Interestingly, only a few of these genes appear to be hypomethylated, with *IL23A* and *IFNGR2* appearing to have the greatest decrease in methylation in BA (S4 Table). Of note, IL23A dimerizes with IL12B to activate IL12 signaling, which does appear to play a role in experimental BA [29]. Our ability to uncover genes previously shown to be important in BA, models of BA, and in cholangiocytes supports the use of the methylation microarray in identifying hypomethylated genes with a potential role in BA pathogenesis.

### Methylation microarray analysis uncovers novel genes upregulated in BA

In addition to genes noted in previous studies on BA, we also identified genes hypomethylated in BA that were not previously reported. The twenty most highly significantly changed probesets are depicted in Table 1, with their most likely associated genes. Several genes on this list (*ARHGEF10*, *ADAP1*, *RAP1GAP*) have a role in intracellular trafficking [19, 32, 33], and we also noted significant changes in *ZEB2* and *PRICKLE4*. Notably, we previously reported that mutations in *ZEB2* are associated with BA, and that morpholino-mediated knockdown of *zeb2* or *prickle1* leads to defects in biliary development [19, 32, 33].

**Fig 3:**
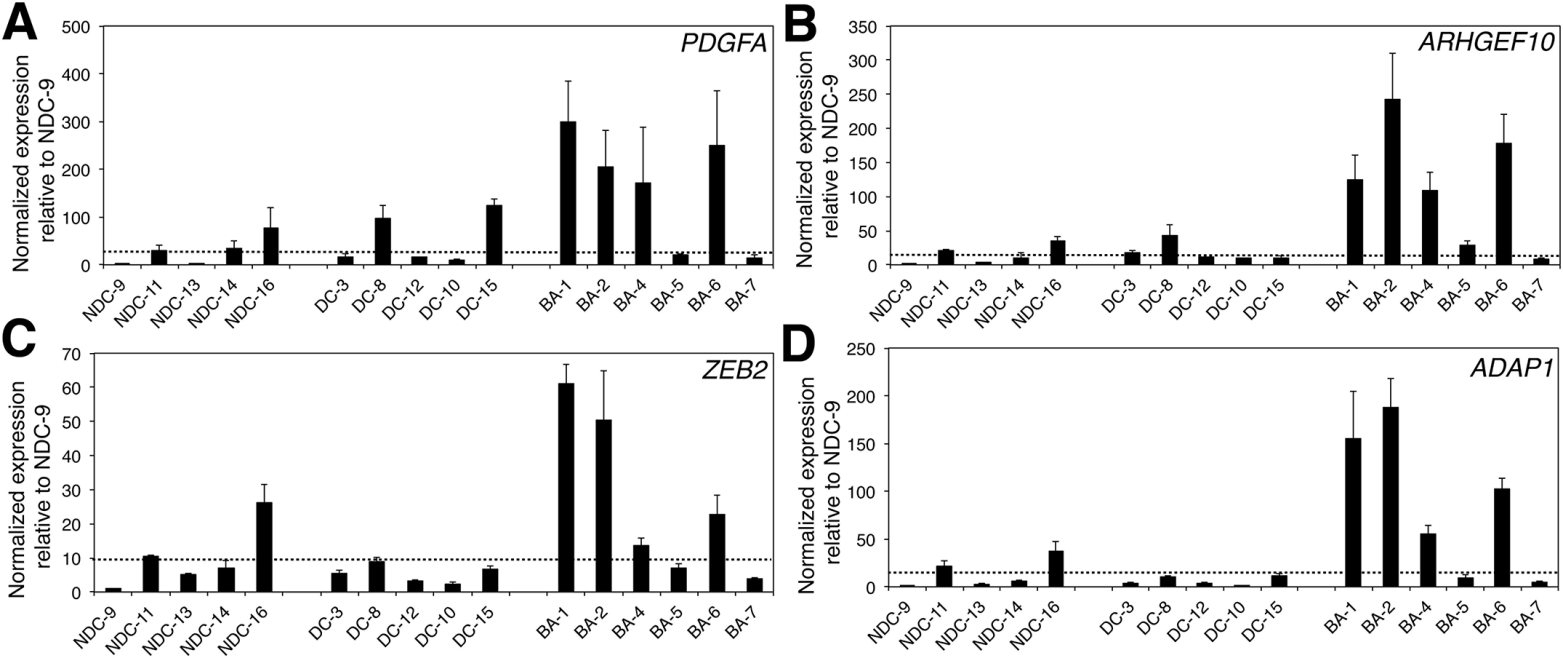
Increased expression of genes identified in methylation microarray analysis. (A-D) Shown are quantitative PCR studies of *PDGFA* (A), *ARHGEF10* (B), *ZEB2* (C), and *ADAP1* (D) expression in liver specimens from non-disease control (NDC), disease control (DC), and biliary atresia (BA) patients. The line represents the average expression level in the NDC samples. (p-values for pooled BA samples vs. NDC, BA vs. DC and BA vs. combined DC and NDC, respectively. *PDGFA*: p<0.05, p≤0.1, p≤0.01; *ARHGEF10*: p<0.05, p<0.05, p<0.005; *ZEB2*: p = 0.18, p<0.1, p<0.05; *ADAP1*: p<0.1, p<0.05, p<0.01).

The decreased methylation of the genes noted in Table 1 would be expected to lead to increased expression of these genes in BA samples. We thus performed quantitative PCR (qPCR) on cDNA derived from samples from NDC, DC, and BA livers (S1B Table). *PDGFA*, *ARHGEF10*, *ZEB2*, and *ADAP1* showed increased expression in BA samples relative to both sets of controls (Fig 3). These findings support the results from the methylation microarray, and also suggest that increased expression of these genes, and potentially others that are hypomethylated in BA, may play a role in pathogenesis in some patients. Interestingly, for reasons that are not entirely clear, two of the BA patients (BA5 and BA7) did not show increased expression of any of the four genes (Fig 3). These two BA subjects were older at the time of liver transplantation (5 and 2 years respectively), compared with the other patients who were all less than 1 year and likely underwent liver transplantation for the indication of persistent cholestasis.

**Fig 4:**
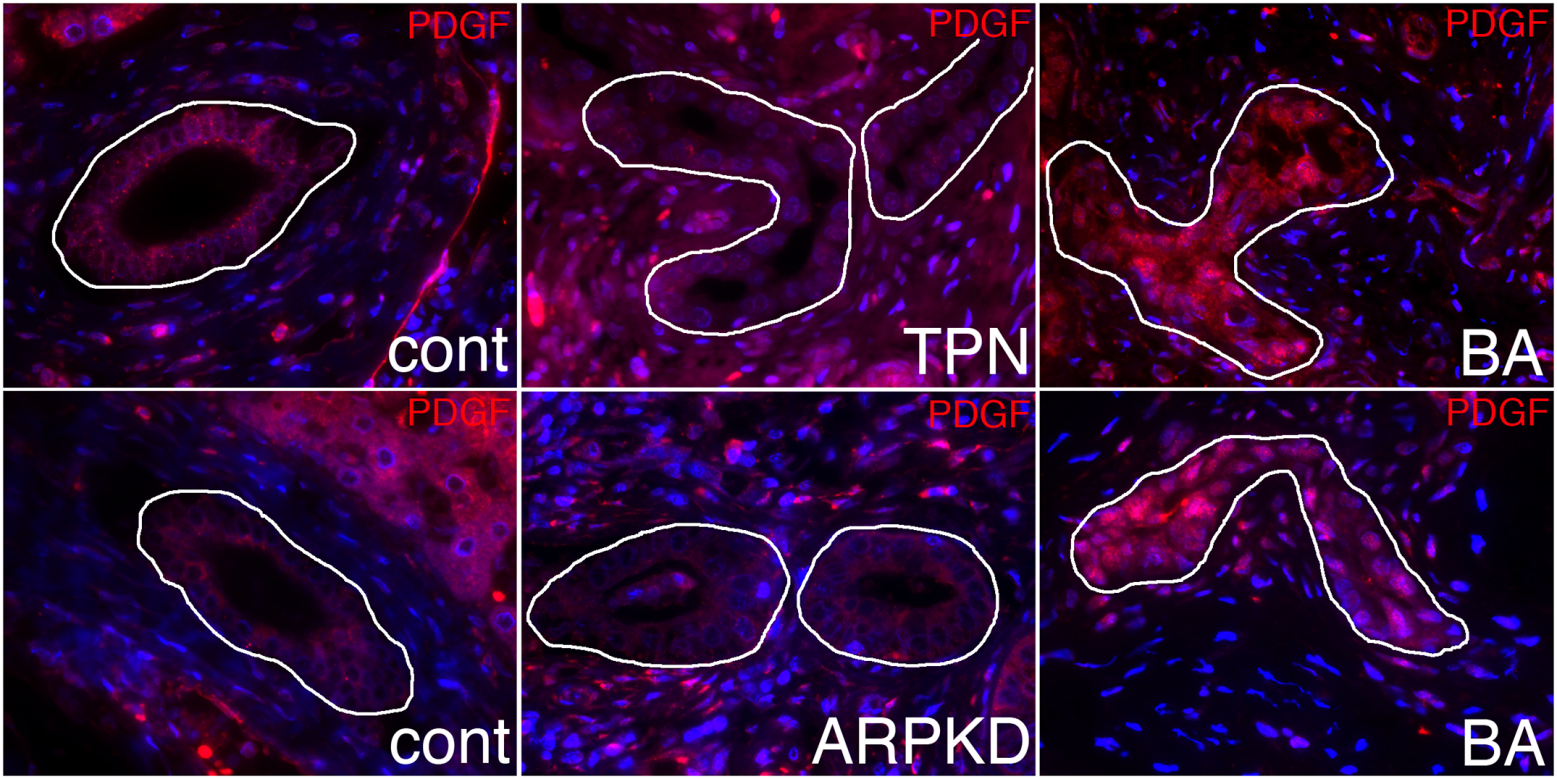
Increased PDGFA in BA samples. Immunostaining of liver biopsy specimens from disease and non-disease control and BA subjects shows increased staining of PDGF in BA cholangiocytes. Duct outlined in white.

The greatest methylation change observed in Table 1 was near the *PDGFA* gene, which encodes the A subunit of platelet-derived growth factor (PDGF). Thus, we examined biopsy specimens from BA patients to determine whether there is increased protein expression of PDGFA in BA patient livers, and to determine the cell specificity. Interestingly, increased PDGFA appears to be specific to cholangiocytes in BA samples (Fig 4). This was unsurprising, as there is fibrosis in many of these samples and PDGF secretion is key to myofibroblast activation and fibrogenesis (reviewed in [34]). These results suggest that there is an increase in PDGFA in cholangiocytes in BA patients, consistent with our findings from the methylation microarray. While such an increase can be mediated by a variety of mechanisms, our methylation microarray results suggest that in BA at least some of this increase may be secondary to a decrease in methylation of *PDGFA*.

Finding genes previously shown to be important in biliary development and BA confirms the importance of DNA hypomethylation as a form of genetic regulation in BA. The ability of our methylation microarray to confirm genes previously identified as being important in BA suggests that the newly identified candidates may be important as well.

### Injection of PDGF-AA leads to biliary defects in zebrafish

To determine whether increased PDGF activity could lead to biliary defects, we injected PDGF peptides into developing zebrafish larvae. We have previously shown that injection of IFNγ or SHH, proteins known to be important in BA, leads to defects in zebrafish biliary development [1, 15]. These defects are manifest by decreased gallbladder uptake of PED-6, a fluorescent phospholipid that concentrates in the gallbladder after ingestion, and by abnormalities in the intrahepatic biliary network as determined by confocal examination of cytokeratin immunostaining of zebrafish livers.

**Fig 5:**
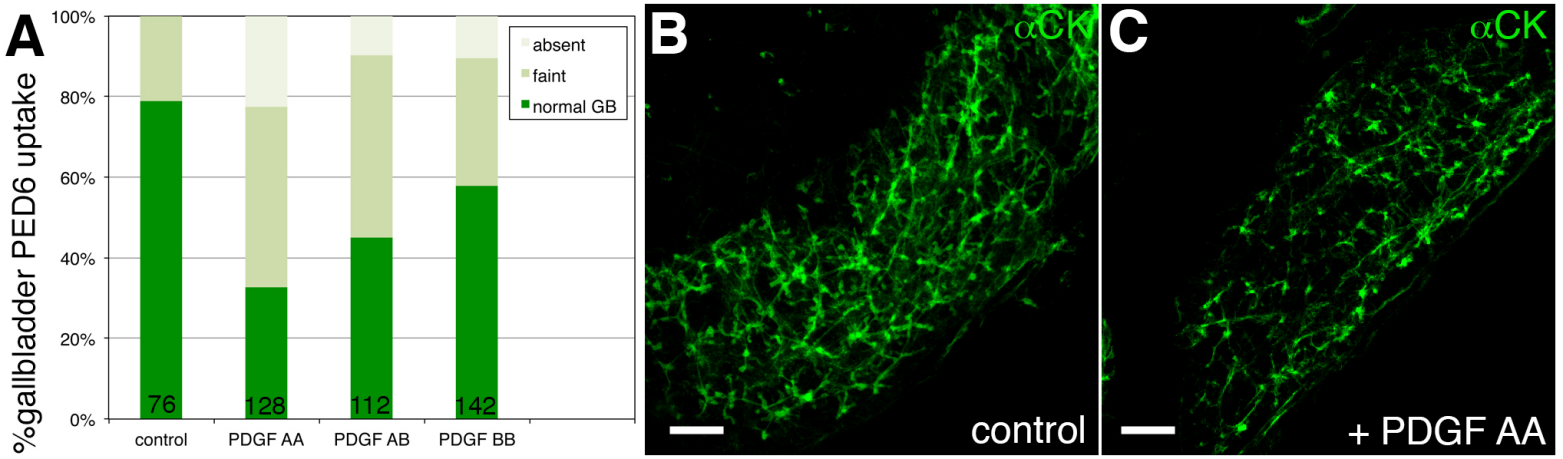
Biliary defects in zebrafish larvae injected with PDGF-AA peptide. **(A)** Larvae were injected with PDGF-AA, PDGF-AB, or PDGF-BB peptide at 2 dpf. PED-6 gallbladder uptake, showing the proportion of larvae at 5 dpf with normal, faint, and absent gallbladders (GB). Note the effect of PDGF-AA on biliary function, as depicted by the increase in faint and absent gallbladders (**p≤ .0001), while the effect of PDGF-AB (p≤.04) and PDGF-BB (p≤.02) is milder. **(B)** Confocal projections of cytokeratin immunostaining of livers from 5 dpf control (cont) and PDGF-AA (PDGF)-injected larvae. The bile ducts are fewer and less complex in the PDGF-injected liver (as quantified in Table 3).

Injection of PDGF-AA, the dimer of PDGFA, leads to impaired gallbladder PED-6 uptake (Fig 5). Both PDGF-AB and PDGF-BB showed modest effects on PED-6 values (p≤0.04 and p≤0.02 respectively) when compared to PDGF-AA injection (p<0.0001) (Fig 5). Supporting the PED-6 findings, livers from larvae injected with PDGF-AA demonstrate abnormalities in intrahepatic ductal anatomy (Fig 5). Injection of PDGF-AA significantly decreased the average number of ducts present in the liver compared to controls (Table 3). These results suggest that increased PDGFA can lead to biliary defects. These studies demonstrate that increased PDGFA expression is sufficient to produce biliary defects, and with our methylation microarray data, are consistent with a model in which hypomethylation of PDGFA contributes to BA pathogenesis.

**Table 3:** Quantification of duct characteristics in zebrafish larvae injected with control vs. PDGF-AA peptide.

	# Ducts	Duct length	# Interconnecting ducts	# Terminal ductules
Control	31.2±6.3	1.69±0.24	9.5±2.4	63.8±27.0
+PDGF-AA	22.5±3.8**	1.53±0.31	2.2±1.6***	36.8±15.0*

For 6 larvae per condition, we have quantified features of the visible ducts in the confocal projections of cytokeratin immunostaining, such as those presented in Fig 5. We have quantified the total number of ducts, their average length (in arbitrary units based on the grid generated by Photoshop), the number of interconnecting ducts and the number of terminal ductules. Interconnecting ducts are defined as ducts in which both ends appear to anastamose with other ducts, while terminal ductules are short ducts that branch off the larger ducts and join to hepatocytes. Note that there is a significant decrease in the total number of ducts (**p<0.05) and the interconnecting ducts (***p<0.005), while the decrease in the number of terminal ductules is marginally significant (*p = 0.05).

### Hedgehog pathway can induce PDGF expression

**Fig 6:**
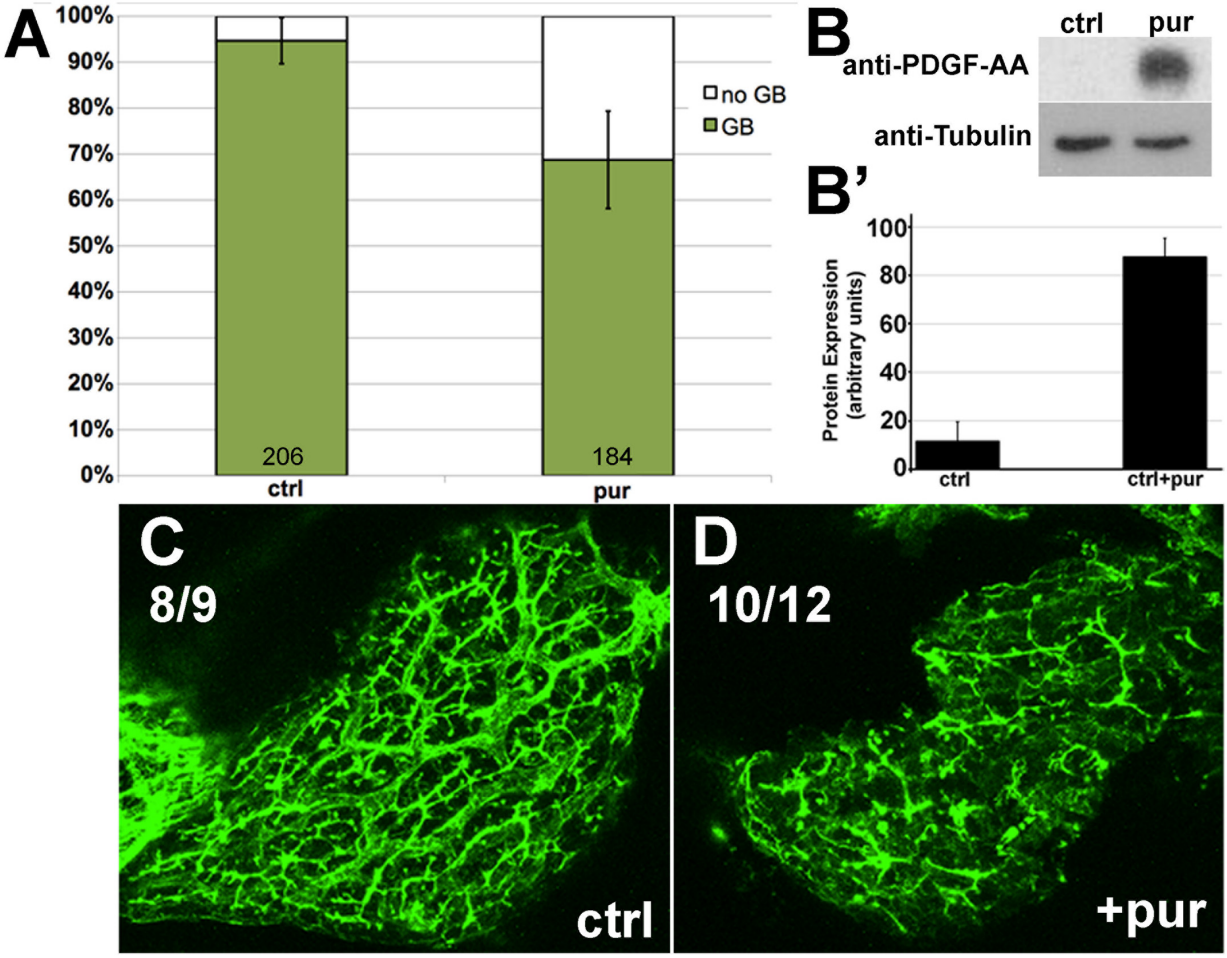
Activation of the HH pathway upregulates PDGF. Larvae were incubated in 30μM purmorphamine, a HH receptor agonist, at 2dpf. (**A**) PED-6 gallbladder uptake shows decreased gallbladder staining in incubated larvae (p≤.00001) (**B**) PDGF-AA protein expression of purmorphamine-incubated larvae relative to the control (ctrl) at 5dpf. (**B’**) PDGF-AA protein quantification of two independent experiments with standard deviations. (**C,D**) Confocal projections of cytokeratin-18 immunostaining of livers from 5dpf control (ctrl) and purmorphamine (pur) incubated larvae. Numbers refer to individuals exhibiting comparable morphology as representative image.

We have shown above that HH pathway genes and *PDGFA* are hypomethylated in BA liver samples, that activation of both pathways leads to biliary defects in an animal model, and that inhibition of the HH pathway rescues defects caused by inhibition of DNA methylation. A previous study reported that PDGF is important in HH signaling by way of primary cilia, through which both HH and PDGF can act [35, 36]. To determine if PDGF can be affected by Hedgehog activity, we examined PDGF expression levels in larvae treated with purmorphamine, which acts as an agonist of Smoothened and thus stimulates HH activity [37]. Larvae treated with purmorphamine demonstrate increased PDGFA expression, in addition to decreased PED-6 uptake and biliary defects apparent by cytokeratin staining (Fig 6). These results demonstrate that increased HH activity leads to impaired biliary function, similar to our previous results, as well as increasing PDGFA protein levels.

## Discussion

Previously, we and others have demonstrated a link between BA and hypomethylation of DNA. Here we provide further evidence linking hypomethylation of liver DNA with BA. We performed methylation microarray analysis on samples from patients with BA and identified multiple genes that are hypomethylated, including several known to be upregulated in BA such as HH pathway genes. We also identified genes not previously known to have a role in BA, and showed increased expression of these genes in BA liver samples. Of these novel genes, *PDGFA* demonstrated the most significant difference in methylation, and our studies showed increased PDGFA expression in BA cholangiocytes. Inhibition of HH signaling in zebrafish larvae treated with a DNA methylation inhibitor resulted in rescue of biliary defects caused by DNA hypomethylation, suggesting that HH activation is downstream of the inhibition of DNA methylation in this model. Injection of PDGF-AA into zebrafish larvae led to developmental biliary defects, while activation of HH signaling increased PDGFA expression in zebrafish larvae. Our findings suggest a potential role for PDGF in mediating biliary defects and possibly in BA, and support the importance of HH activity in BA. These results support a role for DNA hypomethylation in the pathogenesis of BA, possibly via HH and PDGF.

### Epigenetic changes and BA

We have previously reported that BA is associated with decreased DNA methylation in cholangiocytes. Here, we provide further evidence that there is decreased methylation of numerous genes in livers from BA patients, and at least one of these protein products (PDGFA) is increased in cholangiocytes. Others have demonstrated that there is decreased DNA methylation in lymphocytes from BA patients and that this is associated with promoter hypomethylation and increased expression of *IFN*γ [16]. While we did not specifically see *IFN*γ hypomethylation in our array studies, we did see hypomethylation for *IFNGR2* in the BA patient samples.

Several other inflammatory pathways have been implicated as possible mediators in BA, largely based on studies of the RRV-injected mouse model, including IL-12 [29], MCP-1 (*CD46*), MIP-2 *(CXCL2*), TNFα, and complement family members. In our methylation microarray, examination of these gene loci showed methylation changes only near the *IL23A* locus, which is involved in IL12 signaling. While our studies certainly do not rule out an importance of these other pathways, our findings do suggest that IL12/23 activation may at least be more relevant to BA associated with DNA methylation changes. Similarly, our findings support the importance of Hedgehog and TGFβ signaling pathways in BA. Decreased methylation at all of these gene loci may lead to increased gene expression and possible activation of these pathways.

We identified several genes not previously known to be differentially methylated or to have increased expression in BA. We confirmed increased expression of *PDGFA*, *ADAP1*, *ZEB2*, and *ARHGEF10*, suggesting that many of the other differentially methylated genes may have increased expression as well. Because we were able to demonstrate that other genes with a possible role in BA are hypomethylated in our samples, it seems likely that at least some of these newly uncovered genes may be important in BA as well.

### PDGF, Hedgehog, and BA

*PDGF* has not previously been implicated in biliary development or in BA. However, PDGF has been shown to have an important role in vascular development and in fibrogenesis [38]. Additionally, the PDGF receptor is found on primary cilia [36] and in some cell types PDGF appears to be downstream of Hedgehog signaling [39, 40]. Rapid development of fibrosis is a key feature of BA [23], and primary cilia also appear to be important in BA [35] and in other biliary defects such as various types of congenital hepatic fibrosis [41], which share features with BA.

Our studies not only revealed the potential importance of *PDGFA* locus methylation, but also confirmed a potential role of PDGF in mediating biliary defects using a zebrafish model. The importance of PDGF signaling in mediating liver fibrosis supports a potential role for this pathway in BA, despite the pathway not having been previously implicated by analysis of BA specimens or by analysis of the mouse BA model. Thus, overexpression of *PDGFA* from DNA hypomethylation could lead to the excessive fibrosis that is characteristic of BA.

Our observation of developmental biliary defects in zebrafish injected with PDGF-AA supports that PDGF can induce detrimental effects on the developing biliary system. These effects may be on cholangiocytes, or as a result of effects on endothelial cells in the developing zebrafish liver that appear to be critical in mediating polarity of the hepatocyte and consequent biliary development [42]. Thus, a causative role for *PDGFA* overexpression in BA appears plausible.

Additionally, our studies suggest that increased PDGF can result from increased Hedgehog activity. Others have noted that PDGF is a downstream target of Hedgehog in certain cell types. Our studies indicate that PDGF expression can be influenced by both increased Hedgehog activity and decreased methylation in BA. A similar dual regulation is possible for other pathways in BA, as there are reports of Hedgehog signaling being linked to IFNγ [43, 44], Hedgehog and Jagged/Notch working together [45], Jagged/Notch working with TGFβ and SMADs [46], and GLI2 working in the TGFβ pathway [47, 48].

### Possible etiologies for methylation changes in BA

DNA methylation changes seen in BA could arise from an environmental agent, could be a reaction to a causative yet-to-be-identified virus, or perhaps inherited as actual epigenetic changes or as a genetic defect in methylation. There have been several previous studies examining potential causes of BA, including viral infections (CMV, reovirus, others) and environmental changes such as toxins or diet. Several viruses, including viruses that cause liver disease, are known to be associated with DNA methylation changes [49–51]. These changes often allow the virus to remain dormant within the affected cell. Several toxins, including hepatotoxins such as aflatoxin, can induce DNA methylation changes, and dietary changes have clearly been associated with DNA methylation changes. The inability to identify a single virus or other environmental effect in the pathogenesis of BA may reflect that a diverse set of these agents can result in a common final pathway affecting DNA methylation.

Certainly the decreased DNA methylation in BA livers could be secondary, acting more as a reaction to the disease process rather than a cause. Lymphocytes and mesenchymal cells are likely to become activated in BA, and in the process may activate gene expression programs that are noted by methylation changes. While this could be playing a part in the methylation changes we have observed, it seems unlikely to be solely the reason for the methylation changes, as we have observed DNA methylation changes in cholangiocytes, and the changes are fairly specific to BA. One would expect changes in DNA methylation in other conditions, specifically the disease control specimens, if the methylation changes were arising as a reaction to an insult that leads to inflammation and fibrosis. Thus, DNA methylation changes as reaction to other pathogenic insults seems a less likely explanation for our observations.

### Limitations of the current study

Although we were able to identify methylation changes in this study, there are several technical limitations. The differentially methylated genes were identified by a fold change of > 1.2 fold and an uncorrected p-value of < 0.05. Because of the large number of probes on the microarray, these selection criteria could result in false discovery. In addition, the microarray includes multiple probes around each gene of interest, not all of which are hypomethylated. Despite these limitations, additional quantitative PCR studies validated overexpression of the candidate genes of interest, and functional studies indicated an important role for PDGFA in bile duct development and potentially BA pathogenesis. While methylation changes in BA patients have previously been noted in cholangiocytes and in lymphocytes, in the current study we examined all cell types in explanted livers. Such an examination allows us to determine if there is hypomethylation in BA specimens detectable in the whole liver, but it is admittedly difficult to distinguish cell type-specific methylation in this assay. The methylation changes we observed are those that remain apparent despite the mixture of cell types and continued disease progression. In addition, examination of tissue at the time of transplant may miss important changes occurring in the initial stages of the disease. Future studies would ideally examine individual cell types at earlier stages, but such studies may be technically difficult.

## Supporting Information

S1 Table A. Patient samples used for methylation microarray. B. Patient samples used for confirmatory studies.
(DOCX)

S2 Table Primer sequences for Quantitative PCR (qPCR). (DOCX)

S3 Table Methylation of genes identified in microarray studies of BA patients and in studies of *in vitro* biliary development. (DOCX)

S4 Table Hypomethylated inflammatory genes in the methylation microarray. (DOCX)
