# Supplementary material for: Methylation Microarray Studies Highlight PDGFA Expression as a Factor in Biliary Atresia
Source: PLoS One. 2016 Mar 24;11(3):e0151521. doi: 10.1371/journal.pone.0151521 (PMC4806872; doi:10.1371/journal.pone.0151521)
Supplement: S1 Table — A. Patient samples used for methylation microarray. B. Patient samples used for confirmatory studies. (DOCX) [file pone.0151521.s001.docx]

**S1A Table**. **Patient samples used for methylation microarray.**

| **Category** | **Diagnosis** | **Age** | **Sex** |
| --- | --- | --- | --- |
| Non-disease | Urea cycle defect | 2y | F |
| control (NDC) | Urea cycle defect | 5m | F |
|  | Hepatoblastoma (normal tissue) | 20m | F |
| Disease control | Alagille syndrome | 3y | F |
| (DC) | Acute liver failure | 3m | M |
|  | Acute liver failure | 5y | M |
|  | Sclerosing cholangitis | 3y | F |
| Biliary atresia | BA | 11y | F |
| (BA) | BA | 6m | M |
|  | BA | 7m | F |
|  | BA | 2y | M |
|  | BA | 8m | M |
|  | BA | unk | F |

**S1B Table. Patient samples used for confirmatory studies.**

| **Sample** | **Diagnosis** | **Age** | **Sex** |
| --- | --- | --- | --- |
| NDC-9 | Maple syrup urine disease | 2y | M |
| NDC-11 | Propionic acidemia | 2y | M |
| NDC-13 | Urea cycle defect | 11m | F |
| NDC-14* | Urea cycle defect | 2y | F |
| NDC-16* | Hepatoblastoma (adjacent normal tissue) | 20m | F |
| DC-3* | Sclerosing cholangitis | 3y | F |
| DC-8 | Cystic fibrosis | 14y | F |
| DC-12 | Alpha-1-antitrypsin deficiency | 5y | M |
| DC-10 | Sclerosing cholangitis | 3y | F |
| DC-15* | Alagille syndrome | 3y | F |
| BA-1* | Biliary atresia | 8m | M |
| BA-2* | BA | 7m | F |
| BA-4* | BA | 6m | M |
| BA-5 | BA | 5y | M |
| BA-6 | BA | 9m | M |
| BA-7 | BA | 26m | F |
| BA-17 | BA | 7y | F |

Samples denoted with * were used in both studies.

For age, y=year, m=month.
