# Supplementary material for: Methylation Microarray Studies Highlight PDGFA Expression as a Factor in Biliary Atresia
Source: PLoS One. 2016 Mar 24;11(3):e0151521. doi: 10.1371/journal.pone.0151521 (PMC4806872; doi:10.1371/journal.pone.0151521)
Supplement: S2 Table — (DOCX) [file pone.0151521.s002.docx]

**S2 Table.** **Primer sequences for Quantitative PCR (qPCR)**

| **Name** | **Sequence** |
| --- | --- |
| *PDGFA-F* | TGCTTACAGGAAGCCGAGAT |
| *PDGFA-R* | CAGCTTCCTCGATGCTTCTC |
| *ARHGEF10-F* | TCTTCGTGGCTTCGTTTTCT |
| *ARHGEF10-R* | CTCTGGATGGGCTTCATCAT |
| *ZEB2-F* | CGCTTGACATCACTGAAGGA |
| *ZEB2-R* | CTTGCCACACTCTGTGCATT |
| *ADAP1-F* | GACAGAACGAGAGGGTGCTC |
| *ADAP1-R* | GTTACGGGTGCTGTTGTCCT |
| *HPRT-F* | GACCAGTCAACAGGGGACAT |
| *HPRT-R* | CCTGACCAAGGAAAGCAAAG |
