# Supplementary material for: Methylation Microarray Studies Highlight PDGFA Expression as a Factor in Biliary Atresia
Source: PLoS One. 2016 Mar 24;11(3):e0151521. doi: 10.1371/journal.pone.0151521 (PMC4806872; doi:10.1371/journal.pone.0151521)
Supplement: S3 Table — (DOCX) [file pone.0151521.s003.docx]

**S3 Table. Methylation of genes identified in microarray studies of BA patients and in studies of *in vitro* biliary development.**

| **Gene** | **HypoMe in MeMA?** | **NDC meth** | **DC meth** | **BA meth** | **p value BA vs cont** | **probe** | **Study** |
| --- | --- | --- | --- | --- | --- | --- | --- |
| ACVR2A | N |  |  |  |  |  | Chen |
| TM4SF1 | N |  |  |  |  |  | Chen |
| KRT7 | Y | 0.64±0.11 | 0.57±0.13 | 0.45±0.12 | 0.01 | cg22193385 | Chen |
| VIM | N |  |  |  |  |  | Chen |
| UBD | N |  |  |  |  |  | Chen |
| COL1A2 | N |  |  |  |  |  | Chen |
| COL3A1 | N |  |  |  |  |  | Chen |
| COL4A1 | N |  |  |  |  |  | Chen |
| COL6A3 | N |  |  |  |  |  | Chen |
| LUM | N |  |  |  |  |  | Chen |
| SPARC | ± | 0.39±0.05 | 0.36±0.02 | 0.33±0.03 | 0.02 | cg23146663 | Chen |
| CIT | N |  |  |  |  |  | Chen |
| SORCS1 | N |  |  |  |  |  | Chen |
| IGFBP7 | ± | 0.46±0.05 | 0.41±0.08 | 0.36±0.07 | 0.06 | cg19861288 | Chen |
| FOXH1 | N |  |  |  |  |  | Chen |
| TPRKB | Y | 0.26±0.04 | 0.24±0.05 | 0.19±0.04 | 0.01 | cg19861288 | Chen |
| HLA-DRB1 | N |  |  |  |  |  | Chen |
| SCOP | N |  |  |  |  |  | Chen |
| KDSR | N |  |  |  |  |  | Chen |
| RAPGEF2 | N |  |  |  |  |  | Zhang |
| HDAC3 | ± | 0.25±0.02 | 0.23±0.04 | 0.19±0.03 | 0.03 | cg11153694 | Zhang |
| CCNA2 | N |  |  |  |  |  | Zhang |
| MBD4 | N |  |  |  |  |  | Zhang |
| IGF2 | N |  |  |  |  |  | Zhang |
| SPRY4 | N |  |  |  |  |  | Zhang |
| WSB1 | N |  |  |  |  |  | Zhang |
| GLI2 | ± | 0.79±0.06 | 0.76±0.07 | 0.68±0.06 | 0.03 | cg12080831 | Zhang |
| SH2D3C | ± | 0.65±0.05  0.18±0.03 | 0.63±0.06  0.18±0.08 | 0.56±0.06  0.12±0.06 | 0.01  0.04 | cg12080831  cg24545967 | Zhang |
| RUNX1 | Y | 0.52±0.10 | 0.49±0.12 | 0.34±0.10 | 0.01 | cg11737478 | Zhang |
| TDGF1 | ± | 0.76±0.04  0.67±0.03 | 0.77±0.05  0.70±0.04 | 0.66±0.04  0.59±0.04 | 0.004  0.01 | cg02413040  cg20941258 | Zhang |
| BMP1, 8A | N |  |  |  |  |  | Ader |
| SMAD3 | ± | 0.70±0.07 | 0.67±0.11 | 0.57±0.09 | 0.04 | cg23009419 | Ader |
| PSEN1, 2 | N |  |  |  |  |  | Ader |
| LFNG | ± | 0.64±0.08  0.50±0.04 | 0.57±0.10  0.43±0.08 | 0.48±0.09  0.38±0.07 | 0.04  0.02 | cg00604410  cg06804091 | Ader |
| CXCL10 | N |  |  |  |  |  | Leonhardt |
| CCL5 | N |  |  |  |  |  | Leonhardt |
| IFNG | N |  |  |  |  |  | Dong |

Note that only a handful of these genes appear to have significant differences in methylation between BA and control, and that only a minority of these have differences that are likely of any consequence (*KRT7, RUNX1*, *SMAD3, LFNG*).
