# Supplementary material for: Methylation Microarray Studies Highlight PDGFA Expression as a Factor in Biliary Atresia
Source: PLoS One. 2016 Mar 24;11(3):e0151521. doi: 10.1371/journal.pone.0151521 (PMC4806872; doi:10.1371/journal.pone.0151521)
Supplement: S4 Table — (DOCX) [file pone.0151521.s004.docx]

**S4 Table. Hypomethylated inflammatory genes in the methylation microarray.** Probesets associated with inflammatory genes were checked for methylation status in the microarray. Most demonstrated no difference, but notably there was decreased methylation associated with *IFNGR2* and *IL23R*, which have been linked to BA. There were some differences in other genes, as noted.

| **Gene** | **HypoMe in MeMA?** | **NDC meth** | | **DC meth** | **BA meth** | **p value BA vs cont** | **probe** | **Study/Comments** |
| --- | --- | --- | --- | --- | --- | --- | --- | --- |
| IFNGR1 | N |  | |  |  |  |  |  |
| IFNGR2 | Y | | 0.46±0.04 | 0.37±0.13 | 0.24±0.11 | 0.01 | cg22212414 |  |
|  |  | | 0.42+0.04 | 0.37+0.10 | 0.29+0.08 | 0.02 | cg10527943 |  |
|  |  | | 0.57+0.07 | 0.51+0.12 | 0.41+0.10 | 0.01 | cg22669060 |  |
|  |  | 0.60±0.04 | | 0.53±0.09 | 0.43±0.08 | 0.01 | cg24865779 |  |
| IRF2 | ± | 0.68±0.08 | | 0.64±0.10 | 0.55±0.08 | 0.05 | cg17128892 |  |
| C1QA, B, C | N |  | |  |  |  |  |  |
| C1R, S | N |  | |  |  |  |  |  |
| C2 | N |  | |  |  |  |  |  |
| C3 | N |  | |  |  |  |  |  |
| C4A, B | N |  | |  |  |  |  |  |
| C5 | N |  | |  |  |  |  |  |
| C5AR | N |  | |  |  |  |  |  |
| C6 | N |  | |  |  |  |  |  |
| C7 | Y | 0.58±0.01 | | 0.55±0.04 | 0.46±0.03 | 0.02 | cg09179845 |  |
| C8A, B, G | N |  | |  |  |  |  |  |
| C9 | N |  | |  |  |  |  |  |
| CCR1 | N |  | |  |  |  |  |  |
| CCR2 | N |  | |  |  |  |  |  |
| CCR3 | N |  | |  |  |  |  |  |
| CCR4 | N |  | |  |  |  |  |  |
| CCR5 | N |  | |  |  |  |  |  |
| CCR6 | N |  | |  |  |  |  |  |
| CCR7 | N |  | |  |  |  |  |  |
| CCR9 | N |  | |  |  |  |  |  |
| CCR10 | N |  | |  |  |  |  |  |
| CCRL1 | N |  | |  |  |  |  |  |
| CCRL2 | ± | 0.37±0.07 | | 0.36±0.08 | 0.25±0.07 | 0.01 | cg13070763 |  |
| CXCL1-17 | N |  | |  |  |  |  | CXCL2=MIP2 |
| CXCR1-7 | N |  | |  |  |  |  |  |
| CD46 | N |  | |  |  |  |  | MCP1 |
| IL10 | N |  | |  |  |  |  |  |
| IL10RA | N |  | |  |  |  |  |  |
| IL10RB | N |  | |  |  |  |  |  |
| IL11 | N |  | |  |  |  |  |  |
| IL11RA | N |  | |  |  |  |  |  |
| IL12A | N |  | |  |  |  |  |  |
| IL12B | N |  | |  |  |  |  |  |
| IL12RA | N |  | |  |  |  |  |  |
| IL12RB | N |  | |  |  |  |  |  |
| IL13 | N |  | |  |  |  |  |  |
| IL13RA1 | N |  | |  |  |  |  |  |
| IL13RA2 | N |  | |  |  |  |  |  |
| IL15 | N |  | |  |  |  |  |  |
| IL15RA | N |  | |  |  |  |  |  |
| IL16 | N |  | |  |  |  |  |  |
| IL17A-F | N |  | |  |  |  |  |  |
| IL17RA-E | N |  | |  |  |  |  |  |
| IL18 | N |  | |  |  |  |  |  |
| IL18R | N |  | |  |  |  |  |  |
| IL-19 | N |  | |  |  |  |  |  |
| IL-19R | N |  | |  |  |  |  |  |
| IL1A, B | N |  | |  |  |  |  |  |
| IL1R1, 2 | N |  | |  |  |  |  |  |
| IL2 | N |  | |  |  |  |  |  |
| IL20 | N |  | |  |  |  |  |  |
| IL20RA, B | N |  | |  |  |  |  |  |
| IL21 | N |  | |  |  |  |  |  |
| IL21R | Y | 0.57±0.14 | | 0.52±0.12 | 0.41±0.12 | 0.02 | cg02983090 |  |
|  |  | 0.51+0.11 | | 0.52+0.11 | 0.40+0.10 | 0.05 | cg08282819 |  |
| IL22 | N |  | |  |  |  |  |  |
| IL22RA1, A2 | N |  | |  |  |  |  |  |
| IL23A | Y | 0.60±0.08 | | 0.55±0.07 | 0.44±0.08 | 0.01 | cg21477985 |  |
|  |  | 0.67±0.04 | | 0.66±0.06 | 0.55±0.05 | 0.01 | cg19951006 |  |
|  |  | 0.55+0.06 | | 0.52±0.07 | 0.42±0.06 | 0.003 | cg00294382 |  |
|  |  | 0.58±0.05 | | 0.55±0.04 | 0.48±0.04 | 0.02 | cg24773560 |  |
| IL23R | N |  | |  |  |  |  |  |
| IL24 | N |  | |  |  |  |  |  |
| IL25 | N |  | |  |  |  |  |  |
| IL26 | N |  | |  |  |  |  |  |
| IL27 | Y | 0.65±0.07 | | 0.61±0.09 | 0.51±0.08 | 0.02 | cg02195680 |  |
| IL27RA | N |  | |  |  |  |  |  |
| IL28A, B | N |  | |  |  |  |  |  |
| IL28RA | N |  | |  |  |  |  |  |
| IL29 | N |  | |  |  |  |  |  |
| IL2RA, G | N |  | |  |  |  |  |  |
| IL2RB | ± | 0.59±0.06 | | 0.57±0.05 | 0.49±0.05 | 0.03 | cg12335447 |  |
| IL3 | N |  | |  |  |  |  |  |
| IL31 | N |  | |  |  |  |  |  |
| IL31RA | N |  | |  |  |  |  |  |
| IL32 | N |  | |  |  |  |  |  |
| IL33 | N |  | |  |  |  |  |  |
| IL34 | N |  | |  |  |  |  |  |
| IL4 | N |  | |  |  |  |  |  |
| IL4R | ± | 0.51±0.07 | | 0.42±0.16 | 0.35±0.13 | 0.01 | cg16649560 |  |
| IL5 | N |  | |  |  |  |  |  |
| IL5RA | N |  | |  |  |  |  |  |
| IL6 | N |  | |  |  |  |  |  |
| IL6R | N |  | |  |  |  |  |  |
| IL7 | N |  | |  |  |  |  |  |
| IL7R | N |  | |  |  |  |  |  |
| IL8 | N |  | |  |  |  |  |  |
| IL9 | N |  | |  |  |  |  |  |
| TGFB | N |  | |  |  |  |  |  |
| TNF | N |  | |  |  |  |  |  |
| TNFRSF1A, B | N |  | |  |  |  |  |  |
| TNFRSF6B | N |  | |  |  |  |  |  |
| TNFRSF8 | N |  | |  |  |  |  |  |
| TNFRSF9 | N |  | |  |  |  |  |  |
| TNFRSF10A, B, C | N |  | |  |  |  |  |  |
| TNFRSF21 | N |  | |  |  |  |  |  |
| NFKB1 | N |  | |  |  |  |  |  |
| NFKB2 | N |  | |  |  |  |  |  |
